# Single-pulse transcranial magnetic stimulation (sTMS) for the acute treatment of migraine: evaluation of outcome data for the UK post market pilot program

**DOI:** 10.1186/s10194-015-0535-3

**Published:** 2015-06-03

**Authors:** Ria Bhola, Evelyn Kinsella, Nicola Giffin, Sue Lipscombe, Fayyaz Ahmed, Mark Weatherall, Peter J Goadsby

**Affiliations:** eNeura Therapeutics, Sunnyvale, CA USA; Department of Neurology, Royal United Hospital, Bath, UK; Brighton and Sussex University Hospitals, Royal Sussex County, Brighton, UK; Department of Neurology, Hull Royal Infirmary, Hull, UK; Princess Margaret Migraine Clinic, Charing Cross Hospital, London, UK; Headache Group, Basic & Clinical Neuroscience, King’s College London, London, UK; NIHR-Wellcome Trust Clinical Research Facility, King’s College Hospital, London, SE5 9PJ UK

## Abstract

**Background:**

Single pulse transcranial magnetic stimulation (sTMS) is a novel treatment for acute migraine. Previous randomised controlled data demonstrated that sTMS is effective and well tolerated in the treatment of migraine with aura. The aim of the programme reported here was to evaluate patient responses in the setting of routine clinical practice.

**Methods:**

Migraine patients with and without aura treating with sTMS had an initial review (*n* = 426) and training call, and then participated in telephone surveys at week six (*n* = 331) and week 12 during a 3-month treatment period (*n* = 190).

**Results:**

Of patients surveyed with 3 month data (n = 190; episodic, *n* = 59; chronic, *n* = 131), 62 % reported pain relief, finding the device effective at reducing or alleviating migraine pain; in addition there was relief reported of associated features: nausea- 52 %; photophobia- 55 %; and phonophobia- 53 %. At 3 months there was a reduction in monthly headache days for episodic migraine, from 12 (median, 8–13 IQ range) to 9 (4–12) and for chronic migraine, a reduction from 24 (median, 16–30 IQ range) to 16 (10–30). There were no serious or unanticipated adverse events.

**Conclusion:**

sTMS may be a valuable addition to options for the treatment of both episodic and chronic migraine.

## Background

Migraine is the most common cause of disability due to a neurological disorder [1] on a worldwide basis [2]. Migraine can be ameliorated in some patients by life-style advice, and when troublesome, requires treatment of both attacks and strategies to reduce attack frequency with preventives [3]. While much has been determined about the biology of migraine in recent times [4], and the future is promising [5], much needs to be done for the burden on patients and society that migraine brings. A particular issue in migraine is that of side effects, such as weight gain with many preventives [6], or vascular issues with acute attack therapies [7], or both, are driving a need for new effective and well tolerated treatments.

Transcranial magnetic stimulation (TMS) was first described in 1985 [8] and followed seminal work by Merton, Morton and Marsden [9] who had used electrical stimulation of the cortex to dissect questions around the cortical influence on motor reflexes [9–11], and had found the stimulus painful to subjects. sTMS is a non-invasive, safe and painless method of activating the human motor cortex [12]. TMS is based on the principle of electromagnetic induction. A pulse of current passes through a coil located within the device and when the device is placed over a person’s head for a very short duration, it aims to depolarise neurons rapidly within a target area [13]. Given early suggestions of an effect of sTMS in migraine with aura [14], single pulse TMS was studied in cortical spreading depression (CSD) in rat [15], as CSD in rat is considered an excellent model of human aura [16]. sTMS inhibits CSD in rat and cat [15]. It has no effect on nociceptive trigeminocervical neurons [17], while it certainly inhibits nociceptive trigeminothalamic neurons [18, 19].

sTMS has been shown to be effective in acute migraine treatment in patients with migraine with aura in a sham-controlled study [20]. Moreover, it is well accepted to be safe [21]. The device is CE-marked in Europe, so it can be used in clinical practice. The SpringTMS device was introduced to open clinical practice through the post-market pilot program in the UK and migraine patients, both with and without aura, were selected for this treatment. The aim of this program was to assess the impact of sTMS on migraine symptoms and treatment during an initial 3 month treatment period: assessing the effect on pain severity, associated migraine symptoms, attack duration and acute medication use. Patients prescribed treatment from December 2012, were also asked to provide data on disability (HIT-6). We report on the treatment outcomes over a 3 year period, having presented interim outcomes previously in an abbreviated form [22].

## Methods

Twenty specialist headache clinics in the UK participated in the post market pilot program, which commenced in June 2011 following receipt of the CE mark for the SpringTMS device. Five centres, the authors of this report, contributed all but 60 patients.

The headache specialists selected patients in the clinic who had previously found acute medications intolerable (*n* = 89), ineffective or inadequately effective (*n* = 72), or had a medical contraindication (*n* = 57), or some combination of these, to established approaches.

Patients were included who had a diagnosis of episodic migraine with or without aura (*n* = 59) or chronic migraine (*n* = 131) [23]. Patients were excluded if their treating physician felt they were unsuitable, such as presence of metal in the upper body or history of epilepsy.

There were no stipulations placed on patients to change their use of medicines during the TMS treatment period, for those who were also using medicines, except that for the initial experience medication overuse [24] was actively discouraged. Patients could continue on preventives (*n* = 64). Thirty three patients used an anticonvulsant (topiramate *n* = 17; gabapentin *n* = 8; valproate *n* = 3; pregabalin *n* = 2; lamotrigine *n* = 2; topiramate and valproate *n* = 1) with one stopping topiramate 25 mg during the reporting period.

Patients had the option to discontinue device use at any point if they wished.

The data were compiled and analysed with the objective of carrying out a service evaluation that in UK practice does not require Research Ethics Committee review (http://www.hra-decisiontools.org.uk/research/).

### Patient selection

As a non-drug treatment in a specialist clinic setting, sTMS provided an alternative option for a patient group with unmet needs. Throughout the pilot program, participating clinicians selected sTMS specifically for patients with disabling migraine who could not successfully use established treatments for a variety of reasons.

- Lack of efficacy on current treatment.
- Poor tolerability for current acute or preventive treatments.
- Medical conditions that rendered established medications unsafe to use and where patients were trying to conceive.

During the later months of the program patients with medication overuse were included, using combination (Triptan with NSAID ± paracetamol (acetaminophen); *n* = 53), codeine (*n* = 12) or triptans (*n* = 22).

### Device distribution

Following receipt of the doctor’s prescription, the portable device was delivered to the patient at home and within a week, a headache specialist nurse (RB or EK) made first contact with the patient. At the initial call, the patients were advised how to treat, based on the Medical Advisory Board (MAB) guidelines (Table 1). Their typical migraine pattern was documented, in terms of frequency, severity and duration of attacks.

**Table 1:** Initiation strategy from Medical Advisory Board

**•**	Initiate treatment as early as possible when patient first experiences symptoms of migraine, including pain and/or aura symptoms.
**•**	Fill-out the headache diary immediately after treatment or at any time after the migraine attack subsides.
**•**	Record all symptoms, triggers and medications used during each attack in a diary
**•**	Increase the number of pulses delivered during an attack using the following systematic method as needed to improve pain and symptom relief.
To Begin:	
**•**	Encourage the patient to deliver 2 sequential pulses as early as possible at the beginning of the migraine attack.
**•**	Continue with 2 pulses every 15 min for 1–2 h or until pain and symptoms resolve.
**•**	Encourage patients to withhold using rescue medication for the first hour or two if possible.
Evaluate after the first month (3–4 attacks) - if needed increase the number of pulses delivered	
**•**	Encourage the patient to deliver 3 sequential pulses as early as possible at the beginning of the migraine attack.
**•**	Continue with 3 pulses every 15 min for 1–2 h or until pain and symptoms resolve.
Evaluate again after the second month (3–4 attacks) **-** if needed increase the number of pulses delivered	
**•**	Encourage the patient to deliver 4 sequential pulses as early as possible at the beginning of the migraine attack.
**•**	Continue with 4 pulses every 15 min for 1–2 h or until pain and symptoms resolve.

### Data collection

Following a telephone (RB, EK) review with collection of historical baseline data over the previous 3 months, and a training call at the start of treatment, telephone surveys at weeks 6 and 12 were conducted during the treatment period. The questionnaire data at week 12 was anonymised and subsequently analysed. Upon completion, patients’ progress with treatment was reported back to their prescribing doctor.

### Treatment

To treat migraine symptoms, the device is switched on and positioned on the occiput by the patient and the pulse is delivered with the press of a button. The device weighs 1.5 Kg with dimensions H: 81 mm; W: 220 mm; D: 134 mm. A brief sound is heard as the pulse is delivered. A second pulse may be delivered if required. At treatment, a single magnetic field pulse is delivered of nominally 0.9 T [20], measured 1 cm from the device surface, with a rise time of 180 μsec and a total pulse length of less than 1 ms.

**Fig. 1:**
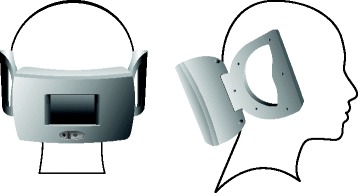
Position of device for treatment

Patients were advised to treat as the guideline (Table 1) and to initiate early treatment where possible. They were advised to place the device over the back of the head (Fig. 1).

Patients commenced treatment by delivering two consecutive pulses (a double pulse), which they repeated after a minimum interval of 15 minutes on treatment days. A total number of 16 single pulses, or eight double pulses, per treatment day could be used with the option to use more if required during months two and three of the treatment period.

They could treat on as many acute migraine days as they wished.

Over time, some doctors advised patients with a frequent migraine pattern to start with daily sTMS treatment and review the effect of varying treatment patterns to reach an optimum individual level.

### Treatment in pregnancy

Three patients were prescribed sTMS during the second trimester of their pregnancy. They each suffered disabling migraine attacks during the first trimester and the attacks continued into the second trimester. They had been treating with medications (paracetamol [acetaminophen] and codeine) without benefit. They each treated as per the guideline (Table 1).

### Analysis

Data were compiled from the patient survey responses into a spreadsheet (Excel 2010) in which summary measures were prepared. Our primary outcome measure was the migraine day. A migraine day was defined as, any day on which there was head pain of moderate or severe intensity, pain scale four or more out of a zero to ten scale, lasting at least 4 hours, and fulfilling current criteria [23]. Secondary measures included a migraine attack, defined as a succession of migraine days terminated by a non-migraine day, and a headache day, which was a day with any headache of any severity for more than an hour. Migraine days at 12 weeks was tested for normality (Kolmogorov-Smirnov Z test). To explore features that may be associated with a useful outcome for migraine prevention a generalized linear model was used with migraine days at 12 weeks as the dependent variable, co-factors: sex, episodic or chronic migraine, aura presence or absence, and covariates of age and baseline migraine days. The link function was identity. Migraine days at 12 weeks was compared to baseline using a Wilcoxon signed-rank test. A 5 % level of significance was used to assess outcomes (IBM SPSS Statistics 21).

## Results

### Patient characteristics

**Fig. 2:**
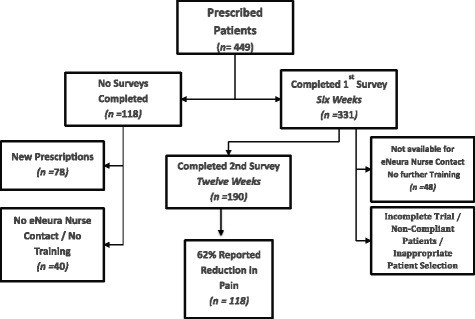
Patient disposition. *New patient- survey provided and no data at the time of the evaluation. **Data set for primary analysis

A total of 449 patients were prescribed sTMS of which 331 completed initial training and first survey at 6 weeks. One hundred and ninety (42 %) used the device to treat migraine attacks for 3 months and completed all surveys (Fig. 2). By the treating physician’s choice, 40 were not available to us for any training or follow-up and without data these are not further reported. These patients returned their devices. Seventy-eight patients are newly prescribed and have not yet completed surveys. An additional 48 patients treated for 3 months but did not complete all surveys. At the time of writing one hundred and ninety (42 %, 140 females), aged 49 ± 13 (mean ± SD) completed questionnaires at the initial contact, 6- and 12-week time points. The report here focuses on the one hundred and ninety patients who treated for 3 months for efficacy and reports adverse events for all patients who have made any such reports (Table 2).

**Table 2:** Patient characteristics

Migraine features	# of patients	# of attacks treated
Migraine with aura	83	3802
Migraine without aura	107	5913
Of these:		
Episodic	59	3470
Chronic	131	6245

### Frequency of migraine days

Patients treated an average of 13 attacks each month, with a baseline frequency of migraine days of 15 (median, 10–20 IQ range). This frequency was 11 [6, 16] at 6 weeks and 8 [3, 13] at 12 weeks. The frequency at 12 weeks was normally distributed (*Z* = 1.13, *P* = 0.16) and reduced compared to the baseline (*Z* = 5.1, *P* < 0.001). The baseline frequencies are set out in Table 3.

**Table 3:** Migraines per month, pain severity and duration of attack by number of patients in each grouping over the reporting period

Migraine days/month	Baseline	6 weeks	12 weeks
<5	8	11	27
5–9	19	35	33
10–14	35	42	45
15–20	56	36	32
21–25	14	12	9
26–30	58	52	44
Pain severity^a^	Baseline	6 weeks	12 weeks
0	0	3	2
1–3	0	44	63
4–6	32	85	75
7–9	140	54	47
10	18	4	3
Duration in days	Baseline	6 weeks	12 weeks
<1	34	66	84
1	55	55	48
2	34	30	27
3	41	24	20
4	19	7	3
>4	2	2	3

^a^Pain severity – 0 to 10 scale

The migraine day outcome could be predicted by a model including presence or absence of aura and baseline frequency (χ^2^_5_ = 93.8, *P* < 0.001). The 12 week frequency was lower in patients with aura (*χ*^2^ = 8.1, *P* = 0.004). Sex, age, or episodic versus chronic diagnosis did not predict the outcome at 12 weeks.

### Pain

Data on one hundred and ninety patients with reports on pain were available. One hundred and eighteen (62 %) patients reported the device was effective at reducing or alleviating their migraine pain after 12-weeks use in over 9000 attacks. At each survey, patients were asked to rate their responses. They rated ‘Good’, ‘Very Good’ and’Excellent’ as effective pain relief and a treatment option they would want to continue using. Patients rating the treatment ‘No Effect’ or ‘Fair’ (*n* = 42), did not find benefit or adequate benefit to continue the treatment.

### Associated symptoms

Of 190 patients reporting at 12 weeks, 174 provided data on at least one associated symptoms- nausea, photophobia or phonophobia. Sixteen reported they did not typically have any associated symptoms. Of the 174 who had such symptoms, 121 (64 %) reported an improvement, defined in their own terms by asking, was there improvement.

### Reduced attack duration

A reduction in the number of headache days per attack was reported in 102 of 185 patients reporting duration data at 12 weeks. The average reduction was a mean decrease from 2.2 days to 0.7 days per attack. Five of the 190 patients did not report duration data at 12 weeks.

**Fig. 3:**
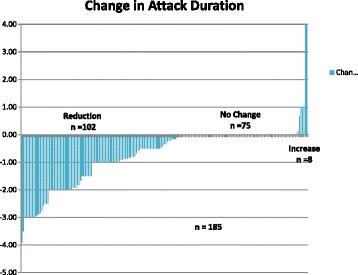
Change in attack duration plotted by patient. While 102 patients had a reduction, 75 had no change and eight had an increase. Five patients did not report the data

A reduction in the number of headache days per attack was reported in 112 (59 %) of 190 patients reporting duration data at 12 weeks (Fig. 3). Forty-eight patients (25 %) reported no change in duration at 12 weeks.

### Dosing schedules

On average patients reported optimal dosing for their symptoms in the range from 10 to 12 pulses per treatment day. The majority of patients, 101 (53 %), treated with two sequential pulses separated by 15-minute intervals, per treatment day. Eight patients preferred a single pulse repeated at 15 min intervals as they derived adequate benefit with this. During the second and third months, 53 patients treated with 3 sequential pulses separated by 15-minute intervals and 28 used 4 sequential pulses separated by 15-minute intervals per treatment day.

### Overall effect

Most patients (120 of 190) reported treating earlier worked better. Patients were not specifically asked but at least one half reported that when their attack was aborted, they felt clearer and did not have lingering ‘mugginess’ and tiredness. Some described that feeling the next day as ‘crystal clear’, which they would not typically experience at the end of an acute attack or when using medications.

### Disability: HIT-6 scoring

One hundred and thirty-nine patients provided pre-treatment scores (66, 62–70; median and interquartile range) and post- (61, 56–66) TMS HIT-6 scores. Of those patients, 19 (14 %) reported no change, 20 (14 %) reported a higher score post-TMS and 100 (72 %) reported a lower score post-TMS. Nineteen (14 %) reported scores below 50 points.

### Medication use

One hundred and sixty four of one hundred and ninety patients reported using acute medications for attacks at baseline. Of these, 119 patients reported a reduction in acute medication use, averaging (8.5 ± 7.7) days reduction in medication use.

### Durability of response

Over the course of the 3 month treatment period, patients who found benefit at 6 weeks, maintained or saw greater improvement by week twelve. At 6 weeks 55 % (*n* = 106) of patients had had benefit and this rose to 62 % (*n* = 118) at 12 weeks.

### Tolerability

sTMS was well tolerated. No serious adverse events were reported. Thirty- eight of 190 (20 %) patients reported transient light-headedness for up to 20 min after pulse delivery. Nineteen reported side effects of either tinnitus, dizziness or tingling over the back of the head at the site of pulse delivery up to 30 min following stimulation. Thirteen reported worsening of migraine symptoms. One patient reported neck and upper shoulder pain that lasted 2 weeks although it is not clear whether this was related to sTMS treatment. The discontinuation rate was 55 % (*n* = 105): no benefit or inadequate benefit (*n* = 49), cost and lack of National Health Service coverage (*n* = 17), inconvenient (*n* = 15), migraine improved or resolved (*n* = 12), inadequate or incomplete trial (did not change dose when suggested; *n* = 7), side effects (*n* = 2) and none stated (*n* = 3).

### Pregnancy

All three patients treated their attacks throughout the pregnancy on a regular basis and reported benefit (Table 4). A reduction in pain severity, shorter attack duration and a reduction in severity of associated symptoms were reported. All three patients subsequently gave birth without complication to healthy children, and continued to treat in the post-partum period. No adverse effects were reported.

**Table 4:** Patients treating during pregnancy

Patient 1	Patient 2	Patient 3
Age: 29. Episodic Migraine with Aura.	Age: 30. Chronic Migraine without aura.	Age: 32. Chronic Migraine without aura.
Pre-pregnancy migraine pattern:	Pre-pregnancy migraine pattern:	Pre-pregnancy migraine pattern:
Frequency: 12 days/month. Duration: 0.5–1 day.	Frequency: 10 days/month plus daily background pain.	Frequency: 15 days/month. Duration: 1–2 days.
Treatment: Triptan and sleep.	No effective treatment.	Treatment: frovatriptan, Syndol (paracetamol [acetaminopheno] + codeine + doxylamine + caffeine) and Naproxen, goes to bed.
During pregnancy:	During pregnancy:	During pregnancy:
Frequency: 2–4 days per week, Duration of 2–3 days, severe and in bed. Estimated 90 % reduced ability to function.	16 days: acute attacks plus daily background pain. Duration: 1 day. Estimated 50 % reduced ability to function.	Treatment: Dihydocodeine during the early pregnancy with partial benefit. Estimated 60 % reduced ability to function.
TMS response: 2 consecutive pulses repeated after 15 min. Consistent reduced pain severity and duration. Could return to function and did not need to go to bed.	TMS response: 4 pulses per day (2 consecutive pulses repeated after 15 min). Stopped attack escalation and reduced the severity back down to a mild tolerable level within 1–2 h. Associated symptoms resolved or did not develop.	TMS response: a single pulse repeated after 15–30 min; up to 4 pulses per attack. Initially combined with dihydrocodeine. Subsequently used sTMS only and could abort the attack within an hour. Associated symptoms rarely developed.

## Discussion

The data presented from an open-label experience with single pulse transcranial magnetic stimulation (sTMS) in a specialist headache clinic setting are broadly consistent with the randomised sham-control data that is available [20]. sTMS seems effective and is well tolerated. The open label data extends the treatment experience to patients with migraine without aura and chronic migraine, doing so based on preclinical data [15, 19], and interestingly the results seem broadly comparable. The data suggest there is a cumulative effect, in that patients do better the longer they are treated, that attacks are shortened, typically by about 1 day and that acute medication use is reduced. From a functional viewpoint, disability scores as recorded with HIT-6 are reduced. Importantly there were no serious or unanticipated adverse events, in keeping with the generally excellent tolerability of the sTMS [21]. Taken together the data support the use of sTMS in migraine.

During the majority of this pilot programme, patients selected were typically those who could not use established acute migraine medications due to intolerable side effects, lack of efficacy or inadequate efficacy or they had medical contraindications to the medicines. Therefore, it was not an aim to look at the impact of sTMS on their medication use and reliance on acute treatments. However, as the programme evolved, patients were prescribed sTMS who also used acute medications regularly. This enabled an additional outcome where many patients reported a reduction in use of acute medicines. This may reflect a combination of factors such as shorter attack duration, reduced number of migraine days and effective pain relief from sTMS. This is a potential significant advantage for sTMS and requires further exploration.

Over time, it also became important to measure the impact of the benefit to patients [25]. An important goal of any treatment is to improve the quality of life for the sufferer. The impact of sTMS on alleviating migraine symptoms also had a significant impact on levels of disability. Reduced attack duration resulted in fewer headache days, less suffering and reduced migraine disability as reflected in HIT-6 scores [26]; HIT-6 is known to be sensitive to change [27]. Adherence to use is another surrogate for effect, since patients tend in general to abandon ineffective therapies. Patients responding to sTMS maintained use, which likely reflects its utility.

There are few safe medication treatments for patients during pregnancy and sTMS may be an option to consider for this patient group [21]. The three patients who treated during pregnancy demonstrated safety and efficacy and derived adequate benefit, and may prove to be an additional treatment option for this patient group. We could not generalise these numbers to a blanket recommendation; suffice to say medical treatment of pregnant, disabled migraine sufferers is very challenging [28] and any new, apparently safe approach needs very serious consideration. In this vein it probably worth considering the TMS load that is offered in the treatment of depression with repetitive TMS (rTMS). A recent meta-analysis lists a range of stimuli from 1 to 2 Hz for 2–5 s with anywhere from 30 to 2,500 pulses per session for between 4 and 20 sessions [29], which is compared in sTMS to at most eight pulses in a day. Taken together with the estimated field strength at the apex of the fundus at term, which is about the same as three exposures to a microwave oven, and is shorter [21], sTMS is worth clinicians’ consideration.

### Limitations

The study is an open-label patient experience without randomisation or allocation by protocol based on the licensed safety of the device and the data that exist. We sought to evaluate the device in practice. There will be a component of the placebo effect which for acute treatment would be between 10 and 25 %, depending on whether there was moderate/severe or mild pain at baseline. For a preventive treatment the placebo effect is assumed to be more substantial, although recent data suggests that may not be the case. The fact that the effect built up may be either a regression to the mean or a true evolution of the treatment effect. The botulinum toxin experience suggests that placebo can be seen over many weeks. Data are certainly missing, as one would expect from clinical practice, and this may have inflated the outcomes. It was a major issue that a substantial group could not be evaluated for logistic reasons of participation, although since this was limited to one site, the impact seems mitigated. Despite all the limitations, the outcomes are generally positive and the therapy very well tolerated.

## Conclusions

The sTMS device has demonstrated safety, efficacy and very good tolerability as an acute migraine treatment in open clinic settings. Our recent analysis suggests there may be a cost advantage to sTMS in the preventive setting [30], and such factors need to be considered as healthcare decisions are made going forward. It thus provided an effective treatment option for patients who could not treat, or treat adequately, with existing treatments. Further clinical use is warranted and careful follow-up will help determine its place in modern therapy.
